# Pinnatoxins’ Deleterious Effects on Cholinergic Networks: From Experimental Models to Human Health

**DOI:** 10.3390/md17070425

**Published:** 2019-07-20

**Authors:** Nicolas Delcourt, Emmeline Lagrange, Eric Abadie, Valérie Fessard, Jean-Marc Frémy, Jean-Paul Vernoux, Marie-Bénédicte Peyrat, Thomas Maignien, Nathalie Arnich, Jordi Molgó, César Mattei

**Affiliations:** 1 Poison Control Centre, Toulouse-Purpan University Hospital and Toulouse NeuroImaging Centre (ToNIC), INSERM1214, Toulouse-Purpan University Hospital, 31059 Toulouse, France; 2 Department of Neurology, Reference Center of Neuromuscular Disease, Grenoble University Hospital, 38000 Grenoble, France; 3 Laboratoire Environnement Ressources du Languedoc-Roussillon, Centre for Marine Biodiversity, Exploitation and Conservation (MARBEC), IRD, Institut Français de Recherche pour l’Exploitation de la Mer (Ifremer), CNRS, Université de Montpellier, CS30171, 34200 Sete Cedex 03, France; 4 Toxicology of Contaminants Unit, ANSES—French Agency for Food, Environmental and Occupational Health & Safety, 35306 Fougères, France; 5 Retired from ANSES—French Agency for Food, Environmental and Occupational Health & Safety, 94701 Maisons-Alfort, France; 6 Research Unit EA 4651 Aliments Bioprocédés Toxicologie Environnements (ABTE), Normandie University, 14000 Caen, France; 7 Risk Assessment Department, ANSES—French Agency for Food, Environmental and Occupational Health & Safety, 94701 Maisons-Alfort, France; 8 Institut des Sciences du Vivant Frédéric Joliot, Service d’Ingénierie Moléculaire des Protéines (SIMOPRO), Commissariat à l’Energie Atomique et aux énergies alternatives (CEA) Saclay, Université Paris-Saclay, F-91191 Gif-sur-Yvette, France; 9 Institut des Neurosciences Paris-Saclay, Centre National de la Recherche Scientifique (CNRS), UMR 9197 CNRS/Université Paris-Sud, F-91198 Gif-sur-Yvette, France; 10 Mitochondrial and Cardiovascular Pathophysiology (MITOVASC), Cardiovascular Mechanotransduction, UMR CNRS 6015, INSERM U1083, Angers University, 49045 Angers, France

**Keywords:** pinnatoxins, cyclic imines, *Vulcanodinium rugosum*, nicotinic acetylcholine receptors, acute neurotoxicity, human intoxication, myasthenia gravis

## Abstract

Pinnatoxins (PnTXs) are emerging neurotoxins that were discovered about 30 years ago. They are solely produced by the marine dinoflagellate *Vulcanodinium rugosum*, and may be transferred into the food chain, as they have been found in various marine invertebrates, including bivalves. No human intoxication has been reported to date although acute toxicity was induced by PnTxs in rodents. LD_50_ values have been estimated for the different PnTXs through the oral route. At sublethal doses, all symptoms are reversible, and no neurological sequelae are visible. These symptoms are consistent with impairment of central and peripheral cholinergic network functions. In fact, PnTXs are high-affinity competitive antagonists of nicotinic acetylcholine receptors (nAChRs). Moreover, their lethal effects are consistent with the inhibition of muscle nAChRs, inducing respiratory distress and paralysis. Human intoxication by ingestion of PnTXs could result in various symptoms observed in episodes of poisoning with natural nAChR antagonists. This review updates the available data on PnTX toxicity with a focus on their mode of action on cholinergic networks and suggests the effects that could be extrapolated on human physiology.

## 1. Introduction

### 1.1. Ecological Aspects

The name pinnatoxin (PnTX) originates from *Pinna attenuata*, a bivalve mollusk from the South China Sea in which these toxic compounds were first collected [1]. Pinnatoxin A (PnTX-A), isolated from the mother-of-pearl *Pinna muricata* harvested in the Japanese island of Okinawa, was the first PnTX with structural elucidation [2]. About a decade ago, a dinoflagellate producing PnTX-E and -F was isolated—but not identified—from water samples of the shallow harbor of Rangaunu in northern New Zealand [3]. These toxins produced neurotoxicity to mice. They belong to the group of cyclic imines, neurotoxic molecules included in the lipophilic toxin group [4]. To date, the dinoflagellate *Vulcanodinium rugosum*, collected from lagoon water samples in southern France, was identified in 2009 as the sole producer of PnTXs [5]. This identification was subsequent to an investigation following an atypical result from the regulatory monitoring of lipophilic toxins in shellfish, showing that mussel extracts from the Ingril lagoon (Mediterranean Sea, France) induced unexpected neurotoxic effects in a mouse bioassay [6]. This observed toxicity was considered unusual and inconsistent with the toxic effects induced by the other known lipophilic toxins [7]. In addition, no microalgal species known to produce neurotoxins was observed in the water samples.

### 1.2. Chemistry of PnTXs

PnTXs belong to the group of cyclic and macrocyclic imines including about 40 different molecules. The acylated esters produced by shellfish detoxification metabolism should also be considered [4]. The cyclic imine group includes toxins with various backbone features: Prorocentrolides, spiro-prorocentrimine, gymnodimines, spirolides, PnTXs, pteriatoxins (PtTX), and portimine. PnTXs and PtTXs share several structural similarities and, among the other compounds, spirolides are structurally the closest to PnTXs [8]. These molecules were found in both plankton and shellfish extracts—mostly bivalves. In shellfish, esterified forms resulting from the metabolic transformation of PnTxs through the formation of acyl esters with fatty acids were also detected [9].

**Figure 1:**
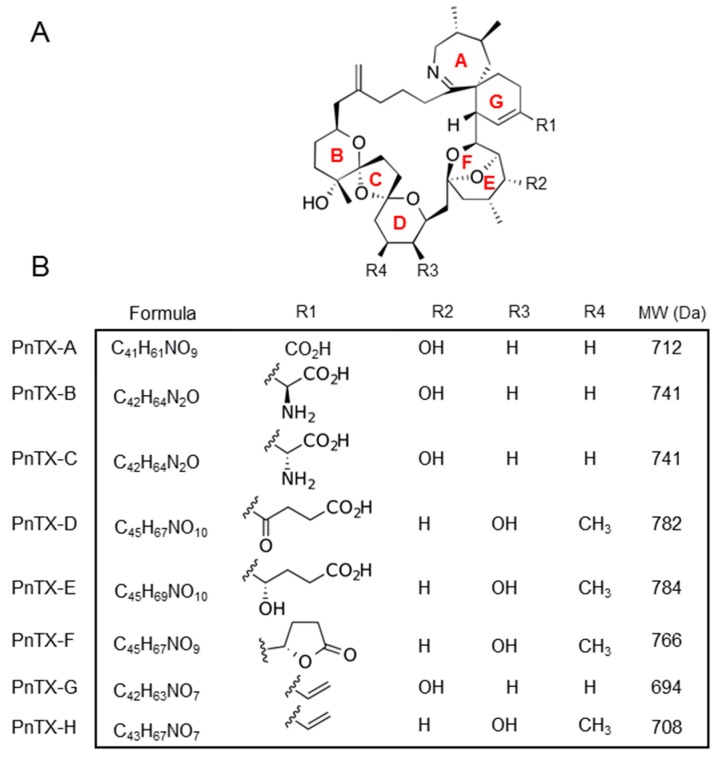
Pinnatoxin (PnTX) chemical features. (**A**) General backbone of PnTXs. Rings in red. The group substitutions R1 to R4 that differ among PnTXs are indicated. (**B**) Chemical structure, formula, and external radicals of PnTX. MW = molecular weight.

PnTXs are characterized by a central invariable backbone with four external substituents R1 to R4 (Figure 1A). This central backbone is an amphoteric macrocyclic structure composed of several rings of type 6,7-spiro (A and G rings), 5,6-bicyclo (bridged E and F rings), or acetal 6,5,6-trispiro (B, C and D rings) [10]. Their molecular weight is about 700 g.mol^−1^ (Figure 1B). The metabolism of PnTXs and PtTXs in shellfish was investigated from environmental samples collected at Franklin Harbor, Australia, with structural toxin elucidation by NMR [8]. PnTX-B, -C, and -D and PtTX-A, -B, and -C were suggested as metabolites of PnTX-E, -F, and -G.

## 2. Acute Toxicity

### 2.1. In Vivo: Symptoms Observed in Mice

Several studies have reported the in vivo effects of purified PnTXs, producing a coherent set of information concerning the acute toxicity of PnTXs. The toxicity in mice occurs rapidly, since the symptoms appear a few minutes after oral or intraperitoneal (i.p) administration [11]. As such, PnTXs can be categorized as fast-acting toxins, like other cyclic imines. Neurotoxic symptoms lead to death by respiratory arrest. Signs of toxicity include decreased mobility—sometimes preceded by a very active phase just after administration—hind limb paralysis, and breathing difficulties [11]. Tremors and jumps are also reported [12]. The lethal dose 50% (LD_50_) in mice varies according to the PnTX analogue and the route of administration. Orally, LD_50_ ranges between 25 and 2800 μg/kg bw for PnTX-F and PnTX-E, respectively (Table 1). Toxicity varies as follows: PnTX-F > PnTX-G ~ PnTX-H >> PnTX-E.

By i.p route, LD_50_ values range between 13 and 115 μg/kg bw, for the PnTX-F and PnTX-A, respectively, and the toxicity can be ranked as follows: PnTX-F > PnTX-G > PnTX-E > PnTX-H > PnTX-A [11,13,14].

At sub-lethal doses, mice recover quickly and completely from PnTX administration without functional consequence [8,11,13]. In fact, the same signs of intoxication are observed but breathing returned to normal within one hour. A lethargic state with piloerection is also described before complete recovery 2 to 3 h after PnTX administration [11]. No macroscopic organ abnormalities are observed at necropsy [8,11,13]. The potent neurotoxicity of PnTXs and associated symptoms led authors to investigate their effects on skeletal muscle contraction. A recent study clearly shows that synthetic PnTX-A and -G, when injected in vivo in the mouse tail muscle at nanomolar concentrations (nmol of PnTX per kg of mouse), caused a dose- and time-dependent reduction of the nerve-evoked compound muscle action potential (CMAP), which reflects the number of muscle fibers that are able to trigger an action potential upon nerve stimulation [15]. The block of neuromuscular transmission in vivo by PnTX-A and -G is reversible in 6 to 8 h.

Methanolic extracts of *V. rugosum* cultures have been also challenged through oral and i.p routes in mice [3,12]. They exhibit a similar acute toxicity to PnTXs with fatal outcome by respiratory arrest. Symptoms preceding death included piloerection, prostration, hypothermia, paralysis of the lower limbs, abdominal breathing, and cyanosis. Death occurred within minutes after mice exposure to the extracts. Post-mortem observations revealed redness in the non-glandular stomach and jejunum together with fluid accumulation in the jejunum [12].

### 2.2. In Vitro Muscle Paralysis

The effects of PnTXs have been also evaluated in isolated rat hemidiaphragms, in order to understand the acute respiratory effects observed in vivo [16]. PnTX-F alone (130–520 nM) or mixed with PnTX-E inhibit the nerve impulse-induced CMAP, without affecting it when evoked by direct muscle stimulation. Neostigmine (8 µM), a well-used inhibitor of acetylcholinesterase, is not able to reverse the inhibition of the CMAP caused by PnTX-F (260 nM) [16]. The PnTX-evoked inhibition of muscle excitability is correlated with the inhibition of muscle contraction [15,16]. Indeed, PnTX-E, -F, and -G drastically reduce muscle contraction of the rat hemi-diaphragm (IC_50_ from 11 to 53 nM). PnTX-F and -G reduce the amplitude of both miniature end-plate potentials (mEPPs)—corresponding to the spontaneous quantal ACh release—and end-plate potentials (EPPs)—corresponding to the evoked quantal ACh release in rat phrenic-cut-hemidiaphragm muscle, with an extracellular medium having a reduced K^+^ concentration [17]. These results, together with those obtained on mouse neuromuscular junctions showing that ACh-evoked potentials, mEPPs, and EPPs were reduced in amplitude and blocked by PnTX-A and PnTX-G in a normal extracellular medium, indicate that PnTXs block the interaction between ACh and its muscle nAChRs at the endplate [15]. Such blockade of neuromuscular transmission in vitro can explain the ability of PnTXs to block the CMAP evoked by motor nerve stimulation in vivo. In addition, preliminary in ovo data indicate an inhibitory effect of PnTX-A on the skeletal development and the muscular apparatus of the chicken embryo [18].

### 2.3. In Vitro: Molecular and Cellular Targets

#### 2.3.1. Nicotinic Acetylcholine Receptors (nAChRs)

The nAChRs, which are the sole targets of PnTXs, are ionotropic receptors acting as cation channels. They are physiologically activated by the neurotransmitter acetylcholine (ACh), in the peripheral and the central nervous system, and can be exogenously activated by nicotine [19]. They are also expressed in non-neuronal tissues [20]. Cholinergic networks implicate nAChRs in different synapses, where they are expressed at presynaptic or postsynaptic sites in order to finetune the neurotransmitter release process, or to activate the post-synaptic cell [21].

**Figure 2:**
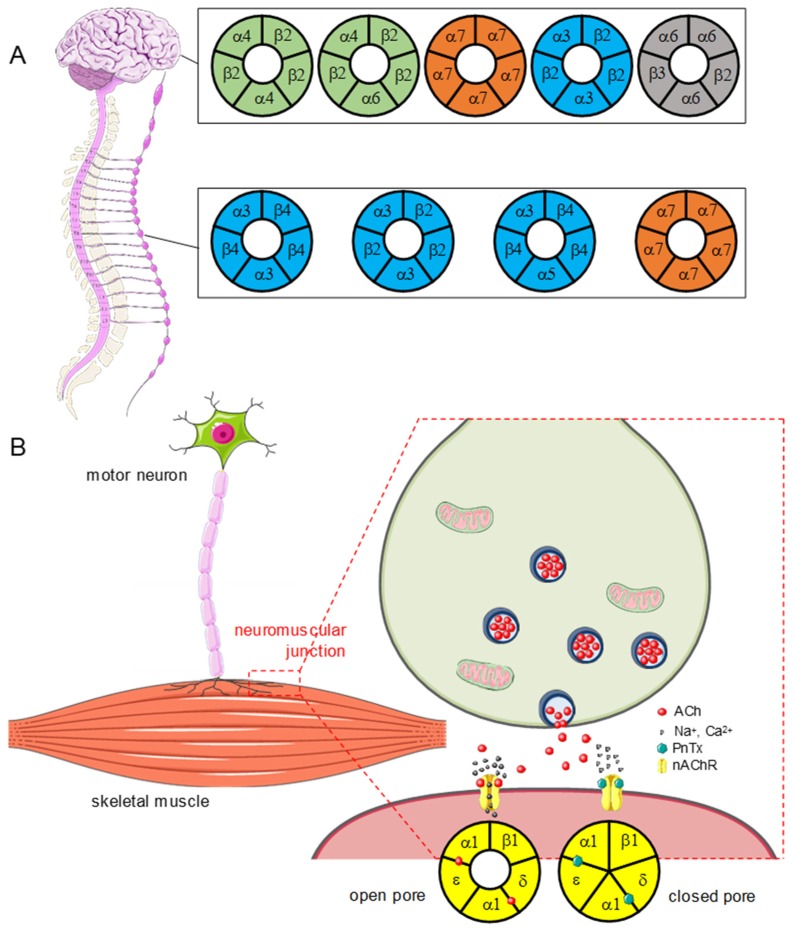
PnTXs act as competitive antagonists of nicotinic acetylcholine receptors (nAChRs). (**A**) Central (upper) and peripheral (lower) nAChRs, as a function of their subunit composition. Some nAChRs are exclusively present in the CNS or in the PNS, for instance the ganglia of the autonomous system. Others, like α7 homopentamers or α3β2 heteropentamers, are expressed in both. Note that only a few of the existing nAChRs are represented (α7β2, α4β2 for example are not presented, see [19,20]). (**B**) Mode of action of PnTXs at the neuromuscular junction. The toxins bind to the sites where it normally binds on the nAChR expressed at the surface of the endplate muscle membrane, thus impairing the opening of the cationic pore. When the receptor is inhibited, it cannot promote the depolarization of the endplate membrane, which impairs the generation of a muscle action potential and thereby the initiation of muscle contraction.

Several types of nAChRs have been characterized. Their differences were initially evidenced by the selectivity of various ligands towards the peripheral vegetative nervous system and the skeletal muscle fibers. The discrepancies in affinity are directly linked to the subunit composition of the receptors, some being homopentamers while others are heteropentamers. As of today, 16 subunits are identified: α1-10, β1-4, γ, δ, and ε – the α8 subunit does not exist in the mammalian nervous system [22]. About 10 different subunit compositions, characteristic of muscular or neuronal expression (Figure 2), have been described.

##### Muscle nAChRs

The nAChRs extracted from the Torpedo electric organ (*Torpedo marmorata*), where their density is high, have been used for pharmacological and physiological characterization [23]. In humans, muscle nAChRs are heteropentamers of type (α1)_2_β1γδ—in the embryo—or (α1)_2_β1εδ—in the adult [24]. These receptors are activated by the binding of two ACh molecules to distinct sites, at the α1-γ, α1-δ, or α1-ε interface. The release of ACh by a motor nerve terminal allows the activation of muscle nAChRs. Their opening induces membrane depolarization, which favors the opening of the skeletal muscle voltage-gated Na^+^ channels (Nav1.4) and the generation of muscle action potentials, which trigger muscle fiber contraction ([25], Figure 2).

##### Neuronal nAChRs

They are heteropentameric receptors combining two α subunits (α2 to α10, but no α8) and three β subunits (β2 to β4), or homopentameric with the α7 subunit. Heteropentamers possess two ACh binding sites, while homopentamers have five. In the central nervous system (CNS), α4, α6, α7, β2, and β3 are the predominant subunits [19]. In the peripheral nervous system (PNS), nAChRs within sympathetic and parasympathetic ganglia express abundantly α3, α5, α7, and β2 and β4 subunits (Figure 2). Activation of central nAChRs results in the opening of the central cation channel and the influx of Na^+^ and Ca^2+^ ions. This post-synaptic membrane depolarization can lead to the genesis of a neuronal action potential, which ends in the release of neurotransmitter at the nerve terminal.

#### 2.3.2. PnTXs Target nAChRs

Like other cyclic imines, PnTXs are competitive antagonists of nAChRs. They bind to ACh binding sites competitively, thus preventing the opening of the pore. The reversibility of this effect depends on the subtypes of nAChR. PnTXs share a common mode of action, and it seems very unlikely that some PnTXs analogues may act differently [26]. PnTXs unambiguously target muscle_type nAChRs causing a concentration-dependent block of muscle contraction induced by nerve stimulation. PnTX-A, -E, -F, and -G block nerve-impulse-evoked muscle contraction, without affecting muscle contraction caused by direct muscle stimulation [15,17]. The muscle paralysis is due to the antagonistic effect of PnTXs on nAChRs (Figure 2).

In fact, PnTX A and G reversibly inhibit nAChRs, but this inhibitory effect is different in muscular and neuronal receptors and may be modulated by the subunit composition of these receptors [27]. These conclusions have been drawn from in vitro electrophysiology experiments using either heterologous expression of nAChRs (cDNA or mRNA) or microtransplantation of purified membranes from the electric organ of Torpedo (*Torpedo marmorata*) in *Xenopus* oocytes using voltage-clamp techniques. To deepen the knowledge on the mode of action of PnTXs, competitive binding and high-affinity radioligand displacement assays were performed on HEK-293 cells transfected with different human or chicken receptor subtypes. Receptors tested were α7 (neuronal), α4β2 and α3β2 (neuronal), and (α1)_2_β1γδ (Torpedo) [4] (Figure 2). Both binding and electrophysiology assays showed that PnTXs bind to muscular and neuronal nAChRs with high affinity, and with a rank order of potency as follows: Muscular receptor (Torpedo) > α7 (human) > α4β2 ~ α3β2. In addition, PnTX selectivity for nAChRs has been demonstrated by testing, using competitive binding assays, on more than 40 other receptors, transporters, and ion channels [26]. In conclusion, muscle nAChRs are not the only targets of PnTXs, but their blocking is enough to experimentally induce a lethal effect. Electrophysiology and binding assays show that these toxins bind to muscle and neuronal nAChRs. Most of the mouse symptoms induced by acute PnTXs exposure can be explained by a blockade of muscle nAChRs.

## 3. Possible Effects to Humans by Extrapolation of Experimental Data

No case of human poisoning due to PnTXs has been reported to date. In China, a human intoxication was first related to the presence of PnTX-A, but finally other contaminants were incriminated [1]. More recently, a *Vulcanodinium rugosum* bloom was identified in Cuba (Cienfuegos Bay), and suspected to have caused skin lesions of swimmers, but PnTXs were not assessed [28]. This observation could stem from the cytotoxicity of one or more of the high number of compounds produced by *V. rugosum*, which, for most of them, remain unidentified [29]. However, it could also be due to PnTXs, since the presence of non-neuronal nAChRs is well documented in keratinocytes [30]. The description of clinical effects observed in humans after exposure to drugs or natural toxins sharing a comparable pharmacology to PnTXs is briefly reviewed hereinafter. Alongside, some pathologies and their symptomatology, which could be expected after exposure to PnTXs, are outlined based on both in vivo and in vitro toxicological studies.

### 3.1. Clinical Effects of nAChR Antagonists

As mentioned previously, PnTXs are nAChR antagonists, targeting peripheral skeletal muscle-type (α1)_2_βγδ and (α1)_2_βεδ nAChRs of the neuromuscular junction, ganglionic α3β2, α3β4, and α7 nAChRs of autonomic ganglia, and the central nervous system α4β2, α7, α3β2, and α4α6β2 nAChR. Antagonists of nAChRs, acting on skeletal muscle or autonomous ganglia, are named muscle relaxants or autonomic agents, respectively, and have been extensively studied. Their main effects are described in the following sections.

#### 3.1.1. Skeletal Muscle Relaxants

The reversible antagonists of muscle nAChRs are used in therapeutics to induce a relaxation of skeletal muscles during surgery intervention procedures. These molecules do not cross the blood–brain barrier. There are two types of muscle relaxants: Depolarizing blocking drugs (DBDs) and non-depolarizing blocking drugs (NDBDs) [31].

The DBDs includes succinylcholine (suxamethonium), which induces a sustained depolarization of the skeletal muscle fibers and desensitizes nAChRs. It is used in surgical anesthesia due to a fast and brief duration of action. DBDs reproduce the effects induced by high doses of ACh without degradation by the synaptic acetylcholinesterase. NDBDs are synthetic molecules, which mimic d-tubocurarine, the main component of curare. This plant toxin has been used as a molecular template for the synthesis of other muscle relaxants. They can be divided in two groups, aminosteroids (pancuronium, vecuronium rocuronium, pipuronium) and benzylisoquinolines (atracurium cisatracurium, doxacurium, mivacurium) [31]. In fact, they act as competitive antagonists, substituting to ACh on nAChRs. As such, they prevent the opening of the nAChR channel and the depolarization of muscle fibers. The effects of NBDBs can normally be reversed by the administration of acetylcholinesterase inhibitor agents, such as neostigmine, as long as some nAChRs remain free for ACh to bind [31].

Side effects of muscle relaxants partly result from their modulation of the muscle nAChRs as well as their ganglionic blocking properties. The nature and intensity of these effects vary for each considered molecule. They mainly affect blood pressure and cardiac rhythm [31].

#### 3.1.2. Autonomic Agents

The autonomic nervous system expresses nAChRs in ganglionic pre- and post-synaptic neurons (Figure 2). The nAChR antagonists exert an inhibition over both divisions—sympathetic and parasympathetic—of the autonomic nervous system and their associated biological effectors. Their global effect is a combination of these inhibitory effects. As such, the observed effect on a given organ depends on the predominance of its sympathetic or parasympathetic innervation. When sympathetic tone predominates, its inhibition will be manifested by parasympathetic effects (i.e., vasodilatation resulting in low blood pressure i.e., hypotension). Conversely, if the parasympathetic tone predominates, ganglionic blocking agents cause sympathetic effects (tachycardia, mydriasis, constipation, urinary retention) [32].

Both natural and synthetic molecules can be used as autonomic drugs, which either activate or inhibit the functions modulated by the sympathetic or parasympathetic nervous systems [33]. That includes antihypertensive drugs, such as hexamethonium and mecamylamine [34]. Their therapeutic use has been limited due to side effects, notably hypotension and tachycardia, possibly accompanied by seizures in case of overdose [35].

Ingestion of anti-cholinergic autonomous agents leads to the following symptoms after 1 to 6 h: Vomiting, dry mouth, constipation, mydriasis, accommodation disorders, elevated intraocular pressure, decreased lachrymal secretion, sinus tachycardia, risk of urinary retention, and acute glaucoma. In case of massive intoxication, excitement, mental confusion, hallucination, hyperthermia, coma, and respiratory depression have been described [36]. Neurotoxicity, sometimes with the development of motor neuron pathologies, is induced after chronic exposure. In addition, teratogenic effects have been reported in animals, as well as in young fed with milk from a goat fed with lupine seed-containing food [37,38].

### 3.2. Human Diseases and Pntx Exposure: Autonomic Dysfunctions?

The involvement of nAChRs in several human disorders has been established and makes this receptor an attractive therapeutic target. Whether implicated in channelopathies resulting from mutations in the nAChR subunit genes, or targeted by autoimmune antibodies, nAChRs are associated with different pathologies affecting the CNS and the PNS [39]. Autoimmune *myasthenia gravis* results from a neuromuscular blockade implicating muscle nAChRs [40,41]. Binding of antibodies to the receptors results in a decrease in the amplitude of the motor endplate potentials, thus preventing the rise of muscle action potentials. The loss of functional nAChRs impairs the nerve-evoked muscle contraction [42,43]. The main symptom of myasthenia is a fluctuating muscle weakness, aggravated by the effort and improved by rest. First and main manifestations are ocular with ptosis and diplopia, but in 80% to 90% of patients, after one year of evolution, other areas are affected, particularly pharyngo-laryngeal muscles and/or limb muscles and/or respiratory muscles. Myasthenia then generalizes [44,45]. Generally, myasthenia gravis is not accompanied by signs of autonomic dysfunction.

Autoimmune myasthenia disease could match with a clinical model of PnTX intoxication. However, autonomic dysfunction may also be the consequence of nAChR blockade by PnTXs especially in the autonomic ganglia—which control the effector organs—and the adrenal medulla, which secretes hormones that act on the cardiovascular system. Autonomic dysfunction symptoms during an experimental exposure to PnTXs were not observed, but we can anticipate that they could predominate in case of human intoxications. In conclusion, the association of autonomic symptoms with neuromuscular signs in humans should challenge the clinician on a possible cause of PnTX intoxication (Table 2). It should, however, be recalled that there are several types of myasthenia, some without antibodies, of presynaptic origin, the majority of which is of genetic origin. In the following table, myasthenia refers to the autoimmune disease, in which antibodies block the muscle nAChRs, paralleling the mode of action of PnTXs. Fatal respiratory distress in humans may not occur as a result of PnTX exposure, as it is well known that the diaphragm is much more resistant to muscle blockers—of NBDB type—than other skeletal muscle groups [46].

## 4. Conclusions

PnTXs are emerging marine biotoxins produced by the dinoflagellate *Vulcanodinium rugosum*. Currently, their presence in seafood is not regulated in Europe nor in any other country in the world. They can be transferred into the food chain: Since 2011, high concentrations of PnTX-G have been observed in mussels from French lagoon every year at summertime and more recently, PnTX-G and -A were detected in mussels from the Atlantic coasts of Spain [47]. PnTX intoxications have not been reported, either because the levels to which consumers were exposed so far were not sufficient to induce severe symptoms, or because humans may be less sensitive than mice to PnTXs. Differences in mouse and human nAChRs might be one reason for this putative discrepancy in species sensitivity.

In mice, PnTXs induce acute neurotoxic effects, within a few minutes after ingestion. Clinical signs of toxicity include decreased mobility, paralysis of the hind legs, tremors, jumps, and breathing difficulties, leading to death by respiratory arrest at high doses. PnTXs are high-affinity nAChR antagonists, which may exert deleterious effects in all neuronal and non-neuronal tissues expressing these receptors. Until now, only acute toxicity studies of PnTXs are available. Further experimental work is needed and should provide knowledge on how PnTXs interact with cholinergic networks—including both neuronal and non-neuronal nAChRs—during both acute and chronic exposures. The toxicokinetics of these toxins and the effects on a broad range of endpoints as a function of the dose and time of exposure should also be investigated. It is very likely that PnTXs may be able to cross most of the biological barriers, including intestinal and blood–brain barriers. PnTXs are classified as fast-acting toxins, which, in case of food poisoning, could provoke deleterious effects on humans. No human poisonings due to PnTX ingestion have been reported to date. Such intoxications have been well documented with other phycotoxins, such as domoic acid, ciguatoxins, or paralytic shellfish toxins [48]. In this view, the French Agency for Food, Environmental, and Occupational Health and Safety recently proposed a provisional acute benchmark value for PnTXs [49]. Based on (i) the clinical signs of toxicity in mice, (ii) the mode of action of PnTXs as nAChR competitive antagonists, and (iii) knowledge of drugs and natural toxins with PnTX-related pharmacology, potential symptoms in humans are proposed here: Muscle weakness (myasthenic-like syndrome), dyspnea, anticholinergic syndrome, dysautonomia, pyramidal syndrome, and seizures. Awareness of healthcare professionals of neurological symptoms induced by PnTXs should help to detect potential cases of human intoxication associated with shellfish consumption.

## Figures and Tables

**Table 1:** Acute in vivo toxicity of PnTXs in mice (oral route). CI_95_: 95% confidence interval. ND: Not determined. *Purity verified by NMR (% not mentioned). MTD: Maximum tolerated dose (dose at which neither mortality was observed, nor clinical signs were evident).

Toxin and % Purity	Route of Administration and Conditions	LD_50_ (µg/kg pc)	MTD (µg/kg pc)	References
PnTX E*	*Per os*	2800	600	[11]
	Fed mice	CI_95_: 2380–3000		
PnTX F*	*Per os*	25.0	9,9	[11]
	Fed mice	CI_95_: 19.1–35.1		
	*Per os*	29.9	ND	
	16 h fasted mice	CI_95_: 25–32		
	Voluntary intake	50.0	16,0	
	Fed mice	CI_95_: 39.4–62.8		
	Voluntary intake	50.0	ND	
	Fed mice	CI_95_: 37.9–71.5		
	Voluntary intake	50.0	ND	
	16 h fasted mice	CI_95_:37.9–71.5		
	Voluntary intake	77.0	ND	
	16h fasted mice	CI_95_: ND		
	Voluntary intake	50.0	ND	
	16h fasted mice	CI_95_: 39.4–62.8		
PnTX-G*	*Per os*	150.0	75	[11]
	Fed mice	CI_95_:105–100		
PnTX-G (100%)	*Per os*	208.0	120	[12]
	3 h fasted mice	CI_95_: 155–281		
PnTX-G*	Voluntary intake	400.0	153	[11]
	Fed mice	CI_95_: 380–470		
PnTX H*	*Per os*	163.0	ND	[13]
		CI_95_: 139–175		

**Table 2:** Clinical signs of toxicity in mice and extrapolation in humans.

Clinical Signs of Toxicity in Mice.	Anatomical or Physiological Support	Possible Outcome in Humans
Loss of motor activity	Neuromuscular junction impairment (skeletal muscles)	Myasthenic syndrome analogous with the disease myasthenia gravis Flaccid paralysis caused by curare
Respiratory depression/arrest	Neuromuscular junction impairment/block (diaphragm)	Respiratory impairment/arrest in myasthenia and myasthenic crisis
Seizure	Central damage via nAChR inhibition	Seizure crisis (impaired GABA release or hereditary mutations)
Leg extension	Spinal interneuron impairment Central impairment	Pyramidal syndrome Babinski sign
Reversibility if no death occurs or after prostigmine injection	Removal of the post-synaptic neuromuscular block	Fluctuation in the degree of myasthenic syndrome or temporary removal of the block with prostigmine Removal of the curare action
Exophthalmos	Increased intraocular pressure	Action of suxamethonium Action of lupin
Hypersalivation, vomiting, diarrhea, bradycardia, bronchoconstriction, miosis	Inhibition of neuronal communication at ganglia synapses	Autonomous agents acting on the sympathetic nervous system (side effects). Nicotinic syndrome
Tachycardia, blood hypertension, mydriasis	Inhibition of neuronal communication at ganglia synapses	Autonomous agents acting on the parasympathetic nervous system (side effects). Muscarinic syndrome

## References

[1] Zheng S., Huang F., Chen S., Tan X., Zuo J., Peng J., Xie R. (1990). The isolation and bioactivities of pinnatoxin. Chin. J. Mar. Drugs.

[2] Uemura D., Chou T., Haino T., Nagatsu A., Fukuzawa S., Zheng S.-Z., Chen H.-S. (1995). Pinnatoxin A: a toxic amphoteric macrocycle from the Okinawan bivalve *Pinna muricata*. J. Am. Chem. Soc..

[3] Rhodes L., Smith K., Selwood A., McNabb P., Van Ginkel R., Holland P., Munday R. (2010). Production of pinnatoxins by a peridinoid dinoflagellate isolated from Northland, New Zealand. Harmful Algae.

[4] Molgó J., Marchot P., Aráoz R., Benoit E., Iorga B.I., Zakarian A., Taylor P., Bourne Y., Servent D. (2017). Cyclic imine toxins from dinoflagellates: A growing family of potent antagonists of the nicotinic acetylcholine receptors. J. Neurochem..

[5] Nézan E., Chomérat N. (2011). *Vulcanodinium rugosum* gen. nov., sp. nov. (dinophyceae): A new marine dinoflagellate from the French Mediterranean coast. Cryptogam. Algol..

[6] Hess P., Abadie E., Hervé F., Berteaux T., Séchet V., Araoz R., Molgó J., Zakarian A., Sibat M., Rundberget T. (2013). Pinnatoxin G is responsible for atypical toxicity in mussels (*Mytilus galloprovincialis*) and clams (*Venerupis decussata*) from Ingril, a French Mediterranean lagoon. Toxicon.

[7] Rossini G.P., Hess P. (2010). Phycotoxins: Chemistry, mechanisms of action and shellfish poisoning. Exp. Suppl..

[8] Selwood A.I., Miles C.O., Wilkins A.L., Van Ginkel R., Munday R., Rise F., McNabb P. (2010). Isolation, structural determination and acute toxicity of pinnatoxins E, F and G. J. Agric. Food Chem..

[9] McCarron P., Rourke W.A., Hardstaff W., Pooley B., Quilliam M.A. (2012). Identification of pinnatoxins and discovery of their fatty acid ester metabolites in mussels (*Mytilus edulis*) from Eastern Canada. J. Agric. Food Chem..

[10] Takada N., Umemura N., Suenaga K., Uemura D. (2001). Structural determination of pteriatoxins A, B and C, extremely potent toxins from the bivalve *Pteria penguin*. Tetrahedron Lett..

[11] Munday R., Selwood A.I., Rhodes L. (2012). Acute toxicity of pinnatoxins E, F and G to mice. Toxicon.

[12] Fessard V., Huguet A., Sosa S., Tubaro A., Aráoz R., Molgó J. (2014). Pinnatoxines en lien avec l’espèce Vulcanodinium rugosum. https://archimer.ifremer.fr/doc/00285/39635/38127.pdf.

[13] Selwood A.I., Wilkins A.L., Munday R., Gu H., Smith K.F., Rhodes L.L., Rise F. (2014). Pinnatoxin H: A new pinnatoxin analogue from a South China Sea *Vulcanodinium rugosum* isolate. Tetrahedron Lett..

[14] Molgó J. (2019). Personal communication.

[15] Benoit E., Couesnon A., Lindovsky J., Iorga B.I., Aráoz R., Servent D., Zakarian A., Molgó J. (2019). Synthetic pinnatoxins A and G reversibly block mouse skeletal neuromuscular transmission *in vivo* and *in vitro*. Mar. Drugs.

[16] Hellyer S.D., Selwood A.I., Rhodes L., Kerr D.S. (2011). Marine algal pinnatoxins E and F cause neuromuscular block in an *in vitro* hemidiaphragm preparation. Toxicon.

[17] Hellyer S.D., Selwood A.I., Rhodes L., Kerr D.S. (2013). Neuromuscular blocking activity of pinnatoxins E, F and G. Toxicon.

[18] Couesnon A., Lindovsky J., Zakarian A., Creuzet S., Molgo J. (2014). Pinnatoxins block skeletal neuromuscular junction activity and affect embryo development. Toxicon.

[19] Gotti C., Zoli M., Clementi F. (2006). Brain nicotinic acetylcholine receptors: native subtypes and their relevance. Trends Pharmacol. Sci..

[20] Zoli M., Pucci S., Vilella A., Gotti C. (2018). Neuronal and extraneuronal nicotinic acetylcholine receptors. Curr. Neuropharmacol..

[21] Dajas-Bailador F., Wonnacott S. (2004). Nicotinic acetylcholine receptors and the regulation of neuronal signalling. Trends Pharmacol. Sci..

[22] Collingridge G.L., Olsen R.W., Peters J., Spedding M. (2009). A nomenclature for ligand-gated ion channels. Neuropharmacology.

[23] Cohen J.B., Weber M., Huchet M., Changeux J.P. (1972). Purification from *Torpedo marmorata* electric tissue of membrane fragments particularly rich in cholinergic receptor protein. FEBS Lett..

[24] Grassi F., Fucile S. (2019). Calcium influx through muscle nAChR-channels: one route, multiple roles. Neuroscience.

[25] George A.L., Komisarof J., Kallen R.G., Barchi R.L. (1992). Primary structure of the adult human skeletal muscle voltage-dependent sodium channel. Ann. Neurol..

[26] Araoz R., Servent D., Molgó J., Iorga B.I., Fruchart-Gaillard C., Benoit E., Gu Z., Stivala C., Zakarian A. (2011). Total synthesis of pinnatoxins A and G and revision of the mode of action of pinnatoxin A. J. Am. Chem. Soc..

[27] Bourne Y., Sulzenbacher G., Radić Z., Aráoz R., Reynaud M., Benoit E., Zakarian A., Servent D., Molgó J., Taylor P. (2015). Marine macrocyclic imines, pinnatoxins A and G: structural determinants and functional properties to distinguish neuronal α7 from muscle α1(2)βγδ nAChRs. Structure.

[28] Moreira A.R., Comas A., Valle A., Seisdedo M., Fernandes L.F. (2016). Bloom of *Vulcanodinium rugosum* linked to skin lesions in Cienfuegos Bay, Cuba. Harmful Algae News.

[29] Geiger M., Desanglois G., Hogeveen K., Fessard V., Leprêtre T., Mondeguer F., Guitton Y., Herve F., Séchet V., Grovel O. (2013). Cytotoxicity, fractionation and dereplication of extracts of the dinoflagellate *Vulcanodinium rugosum*, a producer of pinnatoxin G. Mar. Drugs.

[30] Grando S.A., Horton R.M., Pereira E.F.R., Diethelm-Okita B.M., George P.M., Albuquerque E.X., Conti-Fine B.M. (1995). A nicotinic acetylcholine receptor regulating cell adhesion and motility is expressed in human keratinocytes. J. Investig. Dermatol..

[31] Bowman W.C. (2006). Neuromuscular block. Br. J. Pharmacol..

[32] Wehrwein E.A., Orer H.S., Barman S.M. (2016). Overview of the anatomy, physiology, and pharmacology of the autonomic nervous system. Compr. Physiol..

[33] Becker D.E. (2012). Basic and clinical pharmacology of autonomic drugs. Anesthesia Prog..

[34] Senanayake N., Roman G.C. (1992). Disorders of neuromuscular transmission due to natural environmental toxins. J. Neurol. Sci..

[35] Young J. (2001). Mecamylamine: New therapeutic uses and toxicity/risk profile. Clin. Ther..

[36] Litkey J., Dailey M.W. (2007). Anticholinergic toxicity associated with the ingestion of lupini beans. Am. J. Emerg. Med..

[37] Ortega J.A., Lazerson J. (1987). Anagyrine-induced red cell aplasia, vascular anomaly, and skeletal dysplasia. J. Pediatr..

[38] Panter K.E., James L.F., Gardner D.R. (1999). Lupines, poison-hemlock and *Nicotiana* spp.: toxicity and teratogenicity in livestock. J. Nat. Toxins.

[39] Hurst R., Rollema H., Bertrand D. (2013). Nicotinic acetylcholine receptors: from basic science to therapeutics. Pharmacol. Ther..

[40] Ito Y., Miledi R., Molenaar P.C., Vincent A., Polak R.L., van Gelder M., Davis J.N. (1976). Acetylcholine in human muscle. Proc. R. Soc. Lond. B Biol. Sci..

[41] Vincent A., Newsom-Davis J., Martin V. (1978). Anti-acetylcholine receptor antibodies in d-penicillamine-associated Myasthenia Gravis. Lancet.

[42] Heinemann S., Merlie J., Lindström J. (1978). Modulation of acetylcholine receptor in rat diaphragm by anti-receptor sera. Nature.

[43] Lindstrom J. (1978). How the autoimmune response to acetylcholine receptor impairs neuromuscular transmission in myasthenia gravis and its animal model. Fed. Proc..

[44] Engstrom J.W. (2004). Myasthenia Gravis: Diagnostic Mimics. Semin. Neurol..

[45] Scherer K., Bedlack R.S., Simel D.L. (2005). Does this patient have myasthenia gravis?. JAMA.

[46] Nguyen-Huu T., Molgó J., Servent D., Duvaldestin P. (2009). Resistance to D-tubocurarine of the rat diaphragm as compared to a limb muscle: influence of quantal transmitter release and nicotinic acetylcholine receptors. Anesthesiology.

[47] Lamas J.P., Arévalo F., Moroño Á., Correa J., Muñíz S., Blanco J. (2019). Detection and spatio-temporal distribution of pinnatoxins in shellfish from the Atlantic and Cantabrian coasts of Spain. Toxins.

[48] Ajani P., Harwood D.T., Murray S.A. (2017). Recent trends in marine phycotoxins from Australian coastal waters. Mar. Drugs.

[49] ANSES (2019). Opinion of the French Agency for Food, Environmental and Occupational Health & Safety on the Assessment of the Health Risks Associated with Pinnatoxins in Shellfish. https://www.anses.fr/en/system/files/ERCA2016SA0013EN.pdf.

